# ﻿Revision of the genus *Atholus* Thomson, 1859 (Coleoptera, Histeridae, Histerinae) from the Philippines with additional records

**DOI:** 10.3897/zookeys.1158.100518

**Published:** 2023-04-12

**Authors:** Ian Niel dela Cruz, Masahiro Ôhara

**Affiliations:** 1 Entomological Laboratory, Graduate School of Agriculture, Hokkaido University, N9, W9, Sapporo, 060-8589, Japan; 2 Department of Biology, College of Mathematics and Natural Sciences, Caraga State University, Butuan City, 8600, Philippines; 3 The Hokkaido University Museum, Hokkaido University, N10, W8, Sapporo, 060-0810, Japan

**Keywords:** Coleoptera, genitalia, Histerini, new record, SEM, taxonomy

## Abstract

The Philippine species of the genus *Atholus* Thomson, 1859 are revised and re-examined based on museum as well as freshly collected specimens. *Atholustorquatus* (Marseul, 1854) is re-described, and SEM micrographs and illustrations of both male and female genitalia are provided. *Atholusbakeri* (Bickhardt, 1914) and *Atholusnitidissimus* Desbordes, 1925 are also re-described based on images of syntypes. *Atholuspirithous* (Marseul, 1873) and *A.torquatus* (Marseul, 1854) are new to the Philippine archipelago. *Atholuscoelestis* (Marseul, 1857) and *A.philippinensis* (Marseul, 1854) are provided with diagnostic descriptions and images. A key to the Philippine species is provided.

## ﻿Introduction

*Atholus* Thomson, 1859 is a cosmopolitan genus of Histerinae: Histerini (Coleoptera: Histeridae) spread across the world, with the exception of the Continental Australia and Antarctica. The genus contains 77 described species hitherto; almost half of them occur in the Oriental Region (Mazur 2011).

Philippine *Atholus* have received only limited attention, and in the recent worldwide catalogue of Histeridae (Mazur 2011), only two species – *A.nitidissimus* Desbordes, 1925 and *A.bakeri* (Bickhardt, 1914) from the archipelago were reported as Philippine endemics. Few species were indicated generally in the catalogue to occur in the Oriental region, such as *Atholuscoelestis* (Marseul, 1857); Mazur et al. (2015), however, reported this species from Luzon Island.

Thirteen species of *Atholus* are also currently recorded in Indonesia: *A.tenuistriatus* (Lewis, 1889) from Borneo; *A.crenatifrons* (Lewis, 1899); *A.famulus* (Lewis, 1892); *A.gestroi* (Schmidt, 1897); *A.singalanus* (Marseul, 1880); *A.tetricus* (Lewis, 1902) from Sumatra; and *A.bifrons* (Marseul, 1854); and *A.pinnulae* (Lewis, 1900) reported from both Borneo and Sumatra islands. Moreover, *A.myrmidon* (Marseul, 1862) from Sulawesi and *A.terraemotus* (Lewis, 1900) from Java were also in the checklist, with *A.coelestis* (Marseul, 1857), *A.philippinensis* (1854), and *A.torquatus* (Marseul, 1854), which are also found on these islands. *Atholusbifrons* (Marseul, 1854) was recently reported from Borneo, as well as north towards the Ryukyus islands of Japan (dela Cruz and Ôhara 2022), suggesting that this species might also occur in the Philippines. In addition, *A.bifrons* (Marseul, 1854) was also recorded in Taiwan (Mazur 2008, 2009).

While Ôhara (1992, 1993, 1999b) re-described several Oriental *Atholus* taxa, other species did not receive much attention. This paper provides the first re-description of *A.torquatus* (Marseul, 1854) with illustrations of both male and female genitalia. Additional records, diagnoses, re-descriptions, and figures of all Philippine *Atholus* are provided herein.

## ﻿Materials and methods

Fresh specimens were collected by the senior author under ruminant dung and decaying banana stumps. All museum specimens were loaned from the following institutes: the Hokkaido University Museum, Sapporo (**SEHU**; M. Ôhara), except the syntypes from Muséum National d’Histoire Naturelle, Paris, France (**MNHN**; A. Mantilleri) and Naturhistorisches Museum Berlin, Germany (**MNHUB**; B. Jäger). General observations and dissections were carried out under stereomicroscopes Nikon SMZ745T and Nikon SMZ800. Detailed observations of several structures were performed using SEM (JEOL JSM-6510). Genitalia were dissected and treated according to methods of Ôhara (1994). In this paper, we treat the number of denticles of both apical and outer lateral margins of protibia combined as the 'outer margin', and denticles along the outer lateral margin as the denticles on 'outer sublateral margin', and compare it with the result of Ôhara (1992, 1993). Body measurements are as follows: **PEL** (length between anterior angles of pronotum and apices of elytra), **APW** (width between anterior angles of pronotum), **PPW** (width between posterior angles of pronotum), **EL** (length of elytron along sutural line), and **EW** (maximal width between outer margins of elytra). General morphological terminology follows Ôhara (1994) and Lackner (2010). Regarding syntypes of *A.nitidissimus* Desbordes, 1925 and *A.bakeri* (Bickhardt, 1914), only images of syntypes were available.

## ﻿Systematics

### ﻿Genus Atholus

Taxon classificationAnimaliaColeopteraHisteridae

Thomson, 1859

C53ECDB7-715D-555D-A5A7-9207D23F37F5

Atholus Thomson, 1859: 76 [type species: Histerbimaculatus Linnaeus, 1758: 358, originally designated]; Schmidt 1885: 288; Ganglbauer 1889: 369; Lewis 1906: 402; Bickhardt 1917: 159, 162; 1919: 13, 137, 139; Auzat 1916: 93; Arnett 1962: 378, 381; Halstead 1963: 7, 8; Witzgall 1971: 179, 183; Kryzhanovskij and Reichardt 1976: 382; Mazur 1984: 210, 1997: 128, 2011: 103.
Peranus Lewis, 1906: 401 [type species: Histerscutellaris Erichson, 1834: 151], synonymized by Kryzhanovskij and Reichardt 1976: 384.
Atholister Reitter, 1909: 286 [type species: Histerscutellaris Erichson], synonymized by Heyden, 1910: 317.
Euatholus Kryzhanovskij in Kryzhanovskij & Reichardt, 1976: 387 [type species: Histerduodecimstriatus Schrank, 1781: 39], synonymized by Mazur 1984: 210.

### ﻿Key to the Philippine species of the genus *Atholus* Thomson, 1859

**Table:** 

1	Sutural elytral stria absent	***A.nitidissimus* Desbordes, 1925**
–	Sutural elytral stria present	**2**
2	Dorsal elytral striae 1–3 complete. Dorsal elytral stria 4 present on apical half	***A.philippinensis* (Marseul, 1854)**
–	Dorsal elytral 1–4 striae complete	**3**
3	Apical end of dorsal elytral 3 stria strongly bent inwards. Anterior margin of mesoventrite slightly emarginated	***A.coelestis* (Marseul, 1857)**
–	Apical end of dorsal elytral 3 stria straight, not bent. Anterior margin of mesoventrite outwardly arcuate and no emargination	**4**
4	Lateral pronotal stria not interrupted, connected to anterior marginal stria behind head	***A.pirithous* (Marseul, 1873)**
–	Lateral pronotal stria broadly interrupted in anterolateral angles	**5**
5	Propygidium punctate, punctures becoming finer on pygidium; protibial teeth conspicuous, growing in size apically	***A.torquatus* (Marseul, 1854)**
–	Both propygidium and pygidium strongly punctate	***A.bakeri* (Bickhardt, 1914)**

### ﻿ Atholus philippinensis

Taxon classificationAnimaliaColeopteraHisteridae

(Marseul, 1854)

0EB5F543-ECAF-5E4D-94EC-B6C1959E4B83

**Figures 1–6.**
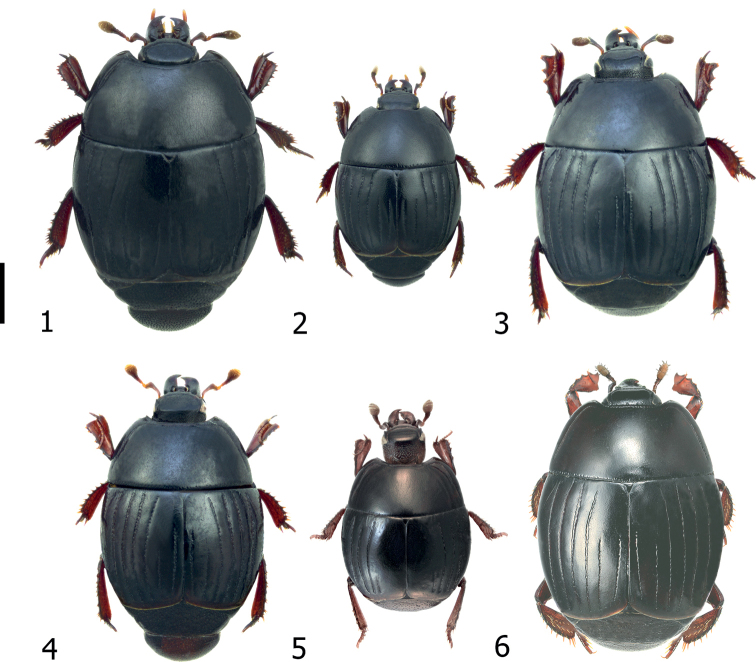
Philippine *Atholus*, dorsal habitus **1***A.philippinensis* (Marseul, 1854) [IC-21-18] **2***A.coelestis* (Marseul, 1857) [IC-21-20] **3***A.torquatus* (Marseul, 1854) [IC-21-49] **4***A.pirithous* (Marseul, 1873) [IC-21-47] **5***A.nitidissimus* Desbordes, 1925 [syntype image] **6***A.bakeri* (Bickhardt, 1914) [syntype image, No. 1639]. Scale bar: 1.00 mm.

**Figures 7, 8.**
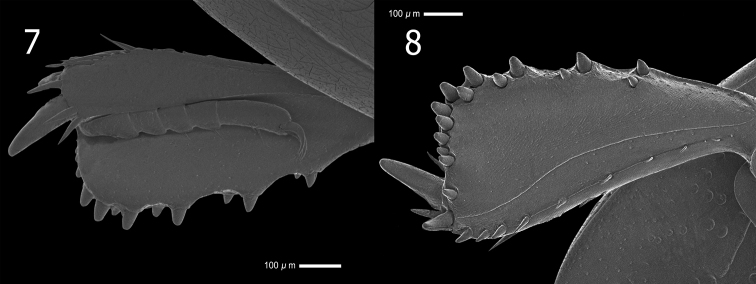
*Atholusphilippinesis* (Marseul, 1854) [IC-21-18] **7** protibia, dorsal view **8** ditto, ventral view.

Figs 1 , 7 , 8 , 35–37

Hister philippinensis Marseul, 1854: 547 [Malaisie (îles Philippines)].
Hister philippensis (sic): Gemminger and Harold 1868: 771.
Hister (Atholus) philippinensis : Bickhardt 1910: 54 [catalogued]; 1913: 173 [Hoozan, Taihorin]; 1917: 194 [catalogued]; Miwa 1931: 57 [Hoozan, Taihorin]; Kamiya and Takagi 1938: 31.
Hister sectator Lewis, 1901: 375, synonymized by Bickhardt, 1917: 194.
Atholus sectator : Lewis 1906: 402.
Atholus philippinensis : Lewis 1906: 402; 1915: 55; Mazur 1984: 215; 1997: 132; 2011: 106 [catalogued]; Ôhara 1999b: 32–36 [Taiwan].

#### Specimens examined.

3 ♂♂, 3 ♀♀. **Mindanao Island**, Agusan del Norte, Butuan, Taligaman, 3 ♂♂, 3 ♀♀ [IC-21-18], 08.56894°N, 125.38534°E 60 m a.s.l., 2021-VI-02 [AN-21-IDC-002], I.N. DELA CRUZ leg.

#### Diagnosis.

*Atholusphilippinensis* (Marseul, 1854) (Fig. 1) is easily distinguished from other Philippine congeners by entire dorsal elytral striae 1–3 (fourth stria is incomplete), and dense punctation of propygidium and pygidium. Among Philippine species, it is the largest one in size, with its markedly wider elytra and posterior angles of pronotum. The number of denticles of the outer sublateral margin of protibia is four.

#### Additional description.

***Female genitalia***: anterior portion of valvifers (Figs 35, 36) paddle-shaped; gonocoxite (Fig. 37) slightly elongate, almost twice as long as broad, shovel-like; inner and outer surfaces differentiated; inner face moderately separated from outer face by elevated lateral ridge; sclerotized setae on apical half of outer face short and somewhat dense; inner face with short and sparse setae; apex of gonocoxite with two teeth; gonostyli present, freely articulated; spermathecae multiple, consisting of four sacs; sacs gradually enlarged and elongate, not sclerotized.

#### Distribution.

Philippines; Malaysia; Indonesia (Sumatra, Borneo, Java); Myanmar, Vietnam; India (Meghalaya); China (Hainan); Taiwan (Mazur 2011).

#### Biology.

This species occurs in decaying banana stumps and are often found along with some species of *Platylister* (Platysomatini, Histerinae, Histeridae).

#### Remarks.

The protibial teeth of *A.philippinensis* (Marseul, 1854) are not as prominent as they are in other species. Moreover, in comparison to the description of Ôhara (1999b), the number of denticles may vary, ranging from 9–11 on the outer margin, one on the inner apical angle, and four or five on the outer sublateral margin. This species was already re-described based on specimens of Taiwan and western Kalimantan, Indonesia (Ôhara, 1999b), including the illustrations of male genitalia and spermatheca of female. Ôhara (1999b) also provided a figure of the spermatheca; we add illustrations of the female gonocoxite and valvifers here (Figs 35–37).

### ﻿ Atholus coelestis

Taxon classificationAnimaliaColeopteraHisteridae

(Marseul, 1857)

C773C507-E3D9-5545-B2E2-24FEB32C0370

Figs 2 , 9–14 , 38–40

Hister coelestis Marseul, 1857: 416, tome, 10, fig. 59 [China].
Hister (Atholus) coelestis : Bickhardt 1910: 53 [catalogued]; 1917: 193 [catalogued]; Desbordes 1919: 399 [Tonkin, Annam, Cochinchine]; 1921: 10 [India]; Kamiya and Takagi 1938: 31 [listed].
Atholus coelestis : Lewis 1906: 402; 1915: 55 [Formosa=Taiwan]; Mazur 1984: 212; 1997: 129; 2011: 104 [catalogued]; Mazur et al. 2015: 1454 [Philippines]; Ôhara, 1992: 173–176; 1994: 137; 1999: 110 [Nansei Islands]; 1999b: 31–32 [Taiwan].
Atholus (Euatholus) coelestis : Hisamatsu and Kusui 1984: 17 [noted, key].
Atholus (Euatholus) coelestes [sic]: Hisamatsu 1985: 228, pl. 41, f. 61 [noted, key, image].
Hister femoralis Motschulsky, 1863: 449, synonymized by Lewis 1885: 465.

#### Specimens examined.

13 ♂♂, 2 ♀♀ and 4 specimens of undetermined sex. **Luzon Island**, Isabela, Angadanan, Pissay, 1 ♂, 16.44207°N, 121.46277°E 60 m a.s.l., 2019-VII-20 [IS-19-IDC-001], I.N. DELA CRUZ leg.; Pangasinan, Asingan, Bantog, 1 ♂, 15.59384°N, 120.41151°E 50 m a.s.l., 2019-VII-22 [PG-19-IDC-001], I.N. DELA CRUZ leg.; Batangas, Calatagan, Balitoc, 1 ♀, 13.51417°N, 120.38138°E 10 m a.s.l., 2019-VI-26 [BG-19-IDC-001], I.N. DELA CRUZ leg. **Mindoro Island**, Oriental Mindoro, Mt. Halcon, 1 ex., 2005-IV. **Panay Island**, Capiz, Dumarao, Bugsuan, 3 ♂♂, 11.14422°N, 122.44405°E 76 m a.s.l., 2019-VIII-03 [CP-19-IDC-001], I.N. DELA CRUZ leg.; Antique, Patnongon, Igbobon, 1 ♂, 1 ex. [IC-21-20], 10.55434°N, 121.59592°E -10 m a.s.l., 2019-VIII-02 [AQ-19-IDC-001], I.N. DELA CRUZ leg.; Iloilo, Calinog, Simsiman, 1 ♂, 11.07008°N, 122.32289°E 70 m a.s.l., 2019-VIII-01 [II-19-IDC-001], I.N. DELA CRUZ leg. **Guimaras Island**, Guimaras, Jordan, Alaguisoc, 1 ♂, 10.37576°N, 122.36379°E 153 m a.s.l., 2019-VII-30 [GU-19-IDC-001], I.N. DELA CRUZ leg. **Negros Island**, Negros Occidental, La Carlota, La Granja, 1 ♂, 10.23566°N, 122.59334°E 90 m a.s.l., 2019-VII-29 [NC-19-IDC-002], I.N. DELA CRUZ leg.; Negros Occidental, Mt. Canlaon, 1 ex., 1988-IV-11-30, D. MOHGAN leg.; Negros Oriental, Tanjay, Azagra, 1 ex., 09.29363°N, 122.08473°E 0 m a.s.l., 2019-VII-31 [NR-19-IDC-001], I.N. DELA CRUZ leg. **Cebu Island**, Cebu, Tuburan, Poblacion, 1 ♂, 10.43204°N, 123.49155°E 15 m a.s.l., 2019-VII-27 [CE-19-IDC-001], I.N. DELA CRUZ leg. **Mactan Island**, Buyong Maribago, Lapu-lapu City, 1 ex., 1996-IV-3, S. SHIMANO leg. **Mindanao Island**, Agusan del Norte, Butuan, Tiniwisan, 1 ♂, 1 ♀ [IC-21-11], 08.57694°N, 125.35521°E 20 m a.s.l., 2021-V-01 [AN-21-IDC-001], I.N. DELA CRUZ leg.; Taligaman, 2 ♂♂, 08.56894°N, 125.38534°E 60 m a.s.l., 2021-VI-14 [AN-21-IDC-003], I.N. DELA CRUZ leg.

#### Diagnosis.

*Atholuscoelestis* (Marseul, 1857) is best characterized by its third dorsal elytral stria extending inwardly towards the apical end of the fourth and fifth striae. The slight emargination on the anterior margin of the mesoventrite is also a distinct character of this species. The number of denticles of the protibia (Figs 13, 14), is 11 on the outer margin, one one the inner apical angle, and eight on the outer sublateral margin. The protibial teeth are slightly prominent only on the outer apical angle, topped with three denticles. The number of denticles on the outer margin may range from 11–13 denticles. The shape of the gonocoxite of *A.coelestis* (Marseul, 1857) is slenderer, becoming narrower towards the apex compared to *A.philippinensis* (Marseul, 1854). Moreover, the presence of a single occipital fovea on the posterior portion of the head of *A.coelestis* (Marseul, 1857) (Fig. 11) is rather a remarkable character differentiating it from other species that has not been previously described.

**Figures 9–14.**
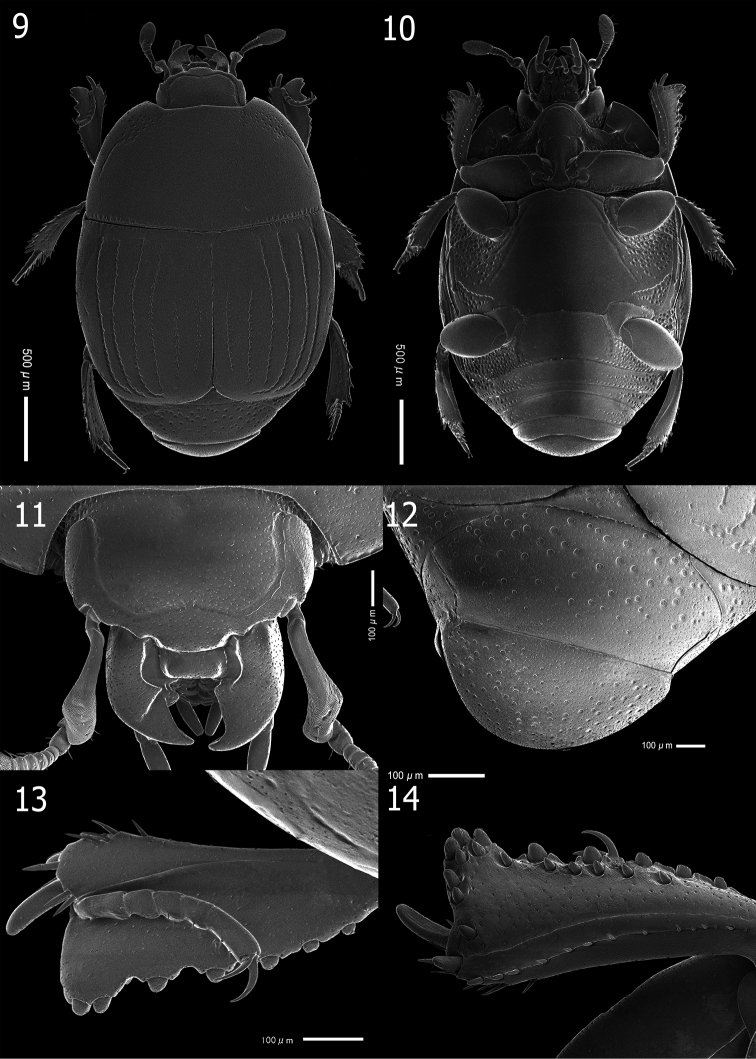
*Atholuscoelestis* (Marseul, 1857) [IC-21-20] **9** habitus, dorsal view **10** ditto, ventral view **11** head, dorsal view **12** propygidium and pygidium **13** protibia, dorsal view **14** ditto, ventral view.

#### Additional description.

***Female genitalia***: anterior portion of valvifers (Figs 38, 39) paddle-shaped; gonocoxite (Fig. 40) elongate, almost 4× as long as broad, not shovel-like, more narrowed on apical end; inner and outer surfaces differentiated; inner face weakly separated from outer face by elevated lateral ridge; sclerotized setae on apical half of outer face short and sparse; inner face with short setae and moderate setae; apex of gonocoxite with two teeth; gonostyli present, freely articulated; spermathecae multiple, consisting of four sacs; sacs gradually enlarged and elongate, not sclerotized.

#### Distribution.

Widely distributed in the Oriental Region including China, Taiwan, Ryukyu Islands (Japan). Also present in the Palearctic Region: Tajikistan and in the Afrotropical Region: Comoros Islands (Mazur 2011).

#### Biology.

All individuals of *A.coelestis* (Marseul, 1857) were collected from dungs of cows and water buffaloes of lowland farms and pastures across all islands of the archipelago. This species may also seem to be moisture-specific, as they were observed to dwell only on more desiccated dungs during field collection.

#### Remarks.

*Atholuscoelestis* (Marseul, 1857) (Fig. 2) is a widespread species across the Philippine archipelago showing a consistent morphology in all individuals examined. *Atholuscoelestis* (Marseul, 1857) was re-described by Ôhara (1992) based on specimens collected from Ryukyu Islands (Japan). Here, SEM micrographs (Figs 9–14) and illustrations of female gonocoxite and valvifers (Figs 38–40) complement Ôhara’s description (1992).

### ﻿ Atholus torquatus

Taxon classificationAnimaliaColeopteraHisteridae

(Marseul, 1854)

18C27BD6-FEC5-5D46-8701-010508D507FF

Figs 3 , 15–20 , 21–28 , 29–34 , 41–43

Hister torquatus Marseul, 1854: 587 [India].
Hister (Atholus) torquatus : Bickhardt 1910: 55 [catalogued]; 1917: 194 [catalogued].
Atholus torquatus : Lewis 1906: 402; Mazur 1984: 218; 1997: 134; 2011: 106 [catalogued]; 2015: 1454.
Hister genuae Lewis, 1888: 639; synonymized by Bickhardt 1913b: 698.
Atholus genuae : Lewis 1906: 402.
Hister mundulus Lewis, 1902: 238; synonymized by Desbordes 1919: 399.

#### Specimens examined.

8 ♂♂, 14 ♀♀ and 7 exs. **Luzon Island**, Bataan, Abucay, Gabon, 8 ♂♂ [IC-21-23], 12 ♀♀ [IC-21-53], 5 exs., 14.42329°N, 120.26222°E 570 m a.s.l., 2019-VII-21 [BA-19-IDC-001], I.N. DELA CRUZ leg.; Laguna, Northern Lucena, Kinabuhayan, 2 ♀♀ [IC-21-49], 1989-II, N. Monreal leg. **Mindoro Island**, Oriental Mindoro, Mt. Halcon, 1 ex., 2005-IV. **Palawan Island**, Puerto Princesa, Barrio Talabigan, 1 ex., 1979-III-24, K. Wada leg.

#### Diagnosis.

*Atholustorquatus* (Marseul, 1854) is recognized with a combination of its interrupted lateral pronotal stria in the anterolateral angle, and fine punctations on the apical portion of its pygidium. This species also possesses remarkable teeth of protibia, increasing in size apically. The structure of the female genitalia of this species is described here for the first time, showing its similarity to the shape of the gonocoxite of *A.philippinensis* (Marseul, 1854), which is broad and shovel-like.

#### Re-description.

**Figures 15–20.**
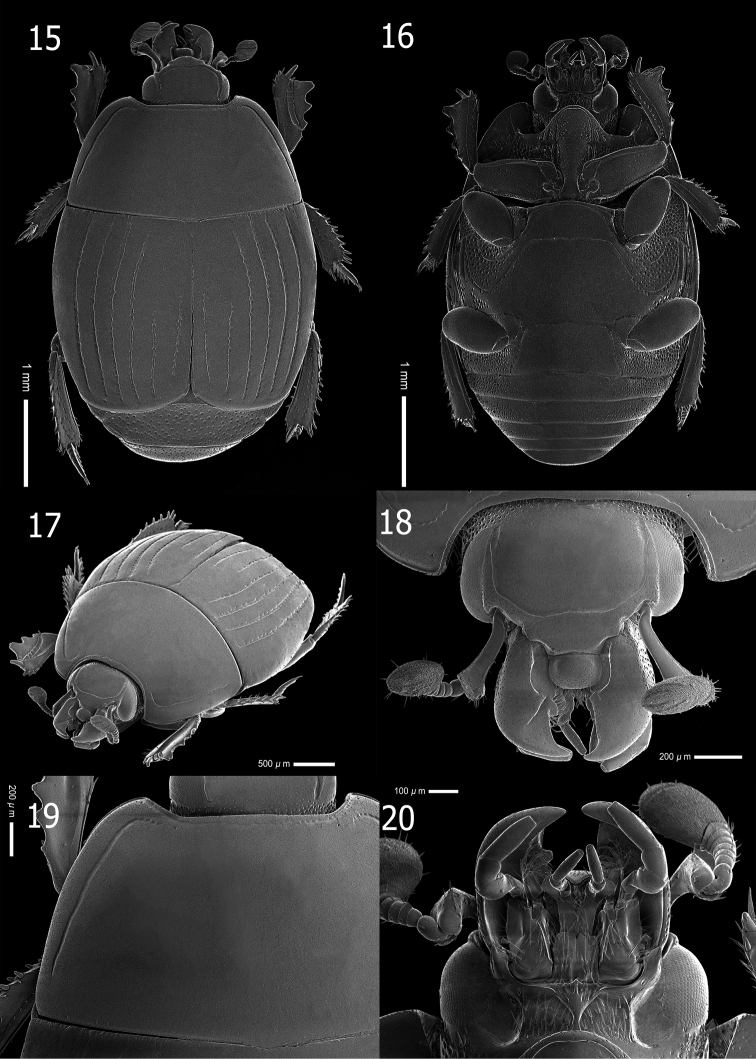
*Atholustorquatus* (Marseul, 1854) [IC-21-23] **15** habitus, dorsal view **16** ditto, ventral view **17** ditto, oblique view **18** head, dorsal view **19** pronotum **20** mouthparts, ventral view.

**Male and female. *Body length***: PEL: 3.13–4.32 mm; APW: 1.11–1.47 mm; PPW: 2.35–2.90 mm; EL: 1.89–2.74 mm; EW: 2.66–3.56 mm. Body (Figs 3, 15–17) oval, moderately convex and black; tibiae, antennae, mouthparts and apical elytral margin rufous.

***Head***: apical margin of clypeus (Fig. 18) short, entire and slightly forward, but anterolateral margin widely crenate; frontal stria rounded, complete and deeply impressed; disk sparsely clothed with fine punctures, separated by 2–3× their diameter; interspaces with alutaceous microsculpture; occipital fovea absent; labrum dorsally finely punctate, raised and transversely long; short labral fringe (Lackner, 2010) present antero-laterally; mandibles covered with fine and even punctures, outer margin rounded, curved inwardly; sub-apical tooth on left mandible large; mandibular apex acutely pointed; eyes large and convex, clearly visible dorsally.

***Pronotum***: marginal pronotal stria laterally complete, continuous onto apical angle and behind head; lateral pronotal stria (Fig. 19) deeply impressed, slightly crenate and complete; lateral stria rather distant from margin, its basal end abbreviated to basal fourth of pronotal length; apical end bent inwardly behind apical angle; anterior pronotal stria absent; disk with sparse microscopic punctures, wholly covered with alutaceous microsculpture; area behind apical angles bare; posterior margin without row of coarse punctures; ante-scutellar region with a single short longitudinal puncture.

**Figures 21–28.**
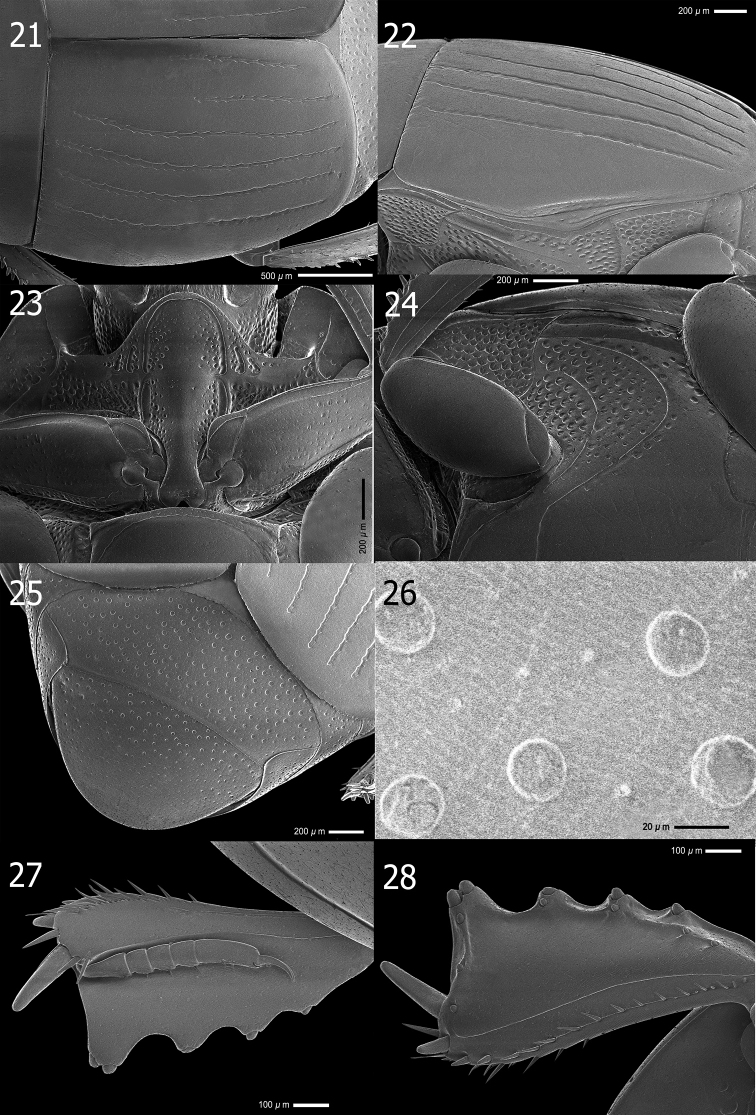
*Atholustorquatus* (Marseul, 1854) [IC-21-23] **21** elytra, dorsal view **22** ditto, oblique view **23** prosternal process **24** meso-and metaventrite **25** propygidium and pygidium **26** propygidium (punctation) **27** protibia, dorsal view **28** ditto, ventral view.

***Elytra***: basal margin with a row of short, longitudinal striae; elytral epipleuron sparsely clothed with fine punctures, with few, coarse punctures on apical half; marginal epipleural stria present on apical half; marginal elytral stria complete, moderately impressed; external subhumeral stria (Fig. 22) generally absent, occasionally noticeable on basal half, abbreviated on basal eighth; internal subhumeral stria absent; oblique humeral elytral stria slightly impressed on basal third; dorsal elytral striae 1–4 (Fig. 21) complete; elytral stria 5 present on apical half; sutural elytral stria abbreviated on basal third; elytral disk covered with sparse, fine punctures, separated by 3–4× their diameter; medio-basal area with alutaceous ground sculpture.

***Propygidium and pygidium***: propygidium (Fig. 26) densely covered with coarse, round and shallow punctures, about 25 µm in diameter, separated by 1–4× their diameter; interspaces with irregular, sparse and fine punctations, separated by 2–3× their diameter; surface with alutaceous sculpture; pygidial punctation (Fig. 25) similar to that of propygidium, coarse punctures of pygidium becoming sparser and finer apically; interspaces with fine punctations.

***Prosternum***: prosternal lobe with anterior margin (Fig. 23) round; medio-apical end of prosternal lobe ascending; marginal prosternal stria deeply impressed, carinate and shortly interrupted medially; short striae present on both baso-lateral corners; lobe with few setiferous coarse punctures inside and outside of marginal stria on both sides, separated by their 1–2× their diameter; disk covered with sparse, finer punctures on apical half; prosternal suture lightly impressed; prosternal process covered with few, setiferous fine punctures; lateral sides descending; lateral prosternal striae deeply impressed and complete; lateral disk with several coarse setiferous punctures; basal half narrow; posterior margin of basal lobe strongly emarginated.

***Meso- and metaventrite***: anterior margin of mesoventrite outwardly arcuate (Fig. 24); marginal mesoventral stria complete, carinate, sparsely crenate; stria behind anterolateral angle present; mesoventral disk sparsely clothed with fine punctures separated by 4–5× their diameter; meso-metaventral suture clearly impressed, complete and medially angulate; lateral metaventral stria deeply impressed, carinate, extending obliquely and posteriorly, united with oblique stria which inwardly extends from basal third of metaventro-metepisternal suture; post-mesocoxal stria extending posteriorly and strongly curved along posterior mesocoxal margin, almost attaining metaventro-mesepimeral suture; punctures of metaventral disk similar to those of mesoventrite; a row of coarse punctures present along inside lateral metaventral stria; longitudinal suture of metaventrite lightly impressed; lateral disk of metaventrite moderately covered with setiferous large round and shallow punctures; interspaces with sparse, coarse to fine punctations; mesepimeron, metepimeron and lateral disk of first abdominal ventrite with dense setiferous, large punctures; interspaces with few coarse to fine punctations; metepisternum with sparse punctures on apical half; punctation of intercoxal disk of first abdominal ventrite similar to that of metaventrite; lateral stria deeply impressed, slightly carinate and complete.

***Legs***: anterior face of protibia (Fig. 27) flattened, dilated and clothed with few, fine ocelloid punctures; basal to median area with weak strigate sculpture; outer lateral margin with four teeth, becoming stronger apically; topped by minute denticles; protarsal groove shallow, with few coarse punctures; anterior protibial stria lightly impressed; inner marginal stria present on basal half, along stria a slightly depressed with row of coarse punctures present; near tarsal insertion with two spine-like tarsal denticles; another one, more distant and longer, located at inner anterior angle; protibial spur moderately long, wider on basal margin, approximately half the length of protarsus; posterior face of protibia (Fig. 28) with sparse, fine punctures and strigate ground sculpture from basal to median surface; number of denticles on outer margin eight, one on inner apical angle, outer sublateral margin three or four; median posterior stria moderately impressed and abbreviated on apical end; inner posterior stria moderately impressed with row of sclerotized setae, terminating in three inner posterior denticles; inner margin of setae present on apical half, with a row of short setae on basal half; inner margin with strigate ground sculpture; profemur sparsely clothed with fine, ocelloid punctations; surface with lightly strigate ground sculpture; marginal stria complete; anterior stria present on apical half; femoral stria almost complete, shortened on basal end; posterior margin with large punctations; a row of setae present on both basal and apical ends.

**Figures 29–34.**
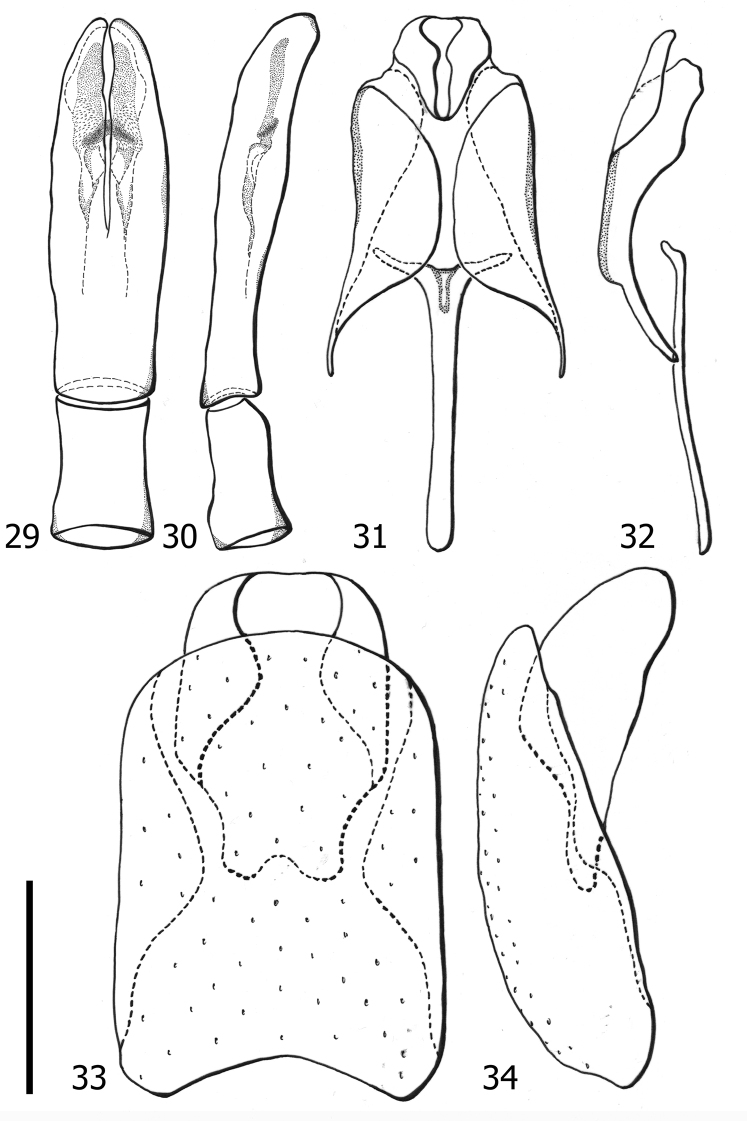
*Atholustorquatus* (Marseul, 1854), male genitalia [IC-21-23] **29** aedeagus, dorsal view **30** ditto, lateral view **31** ninth and tenth tergites, dorsal view **32** ditto, lateral view **33** eighth tergite and sternite, dorsal view **34** ditto, lateral view. Scale bar: 0.50 mm.

***Genitalia***: aedeagus (Figs 29, 30) moderately slender, apically slightly curved ventrad; parameres relatively longer, about as almost as thrice the length of phallobase, slightly fused on basal half; median lobe sclerotized; eighth tergite (Figs 33, 34) entire, with longitudinal fold on both lateral sides; ninth tergite (Figs 31, 32) with lateral folds; tenth tergite dorsally longitudinally divided; spiculum gastrale almost as same length as ninth tergite.

**Figures 35–37.**
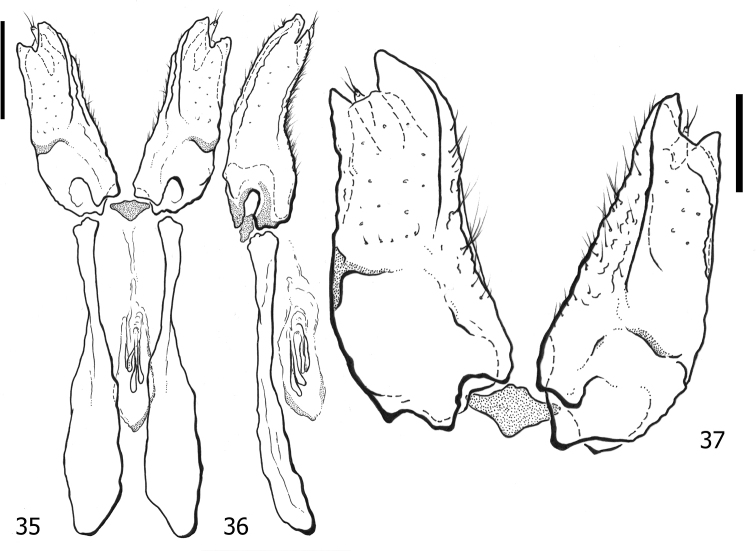
*Atholusphilippinensis* (Marseul, 1854), female genitalia [IC-21-18] **35** dorsal view **36** lateral view **37** dorsolateral view of gonocoxite. Scale bars: 0.20 mm.

**Figures 38–40.**
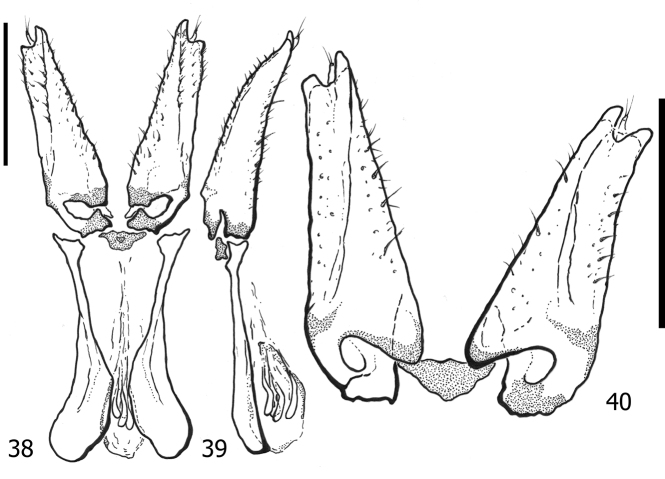
*Atholuscoelestis* (Marseul, 1857), female genitalia [IC-21-11] **38** dorsal view **39** lateral view **40** dorsolateral view of gonocoxite. Scale bars: 0.20 mm.

**Figures 41–43.**
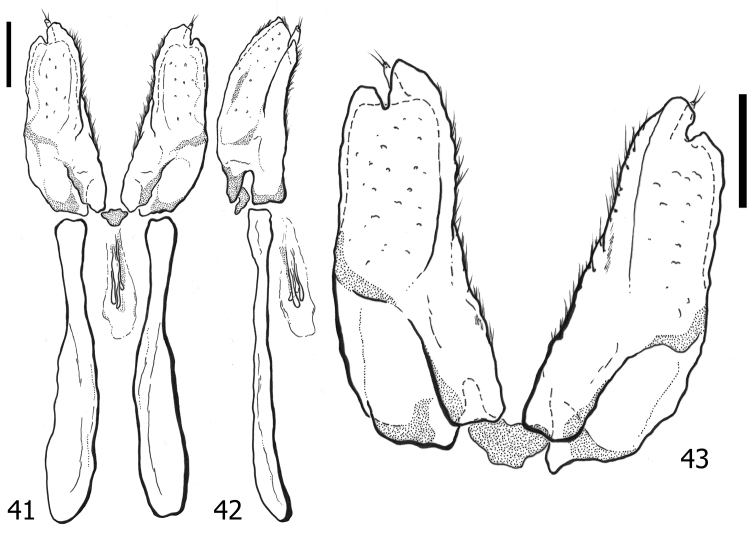
*Atholustorquatus* (Marseul, 1854), female genitalia [IC-21-53] **41** dorsal view **42** lateral view **43** dorsolateral view of gonocoxite. Scale bars: 0.20 mm.

Anterior portion of valvifers (Figs 41, 42) paddle-shaped; gonocoxite (Fig. 43) slightly elongate, almost as twice as long as broad, shovel-like; inner and outer surfaces differentiated; inner face moderately separated from outer face by elevated lateral ridge; sclerotized setae on apical half of outer face short and slightly dense; inner face with short and sparse setae; apex of gonocoxite with two teeth; gonostyli present, freely articulated; spermathecae multiple, consisting of four sacs; sacs gradually enlarged and elongate, not sclerotized.

#### Distribution.

Widespread in the Oriental Region including Indonesia, Myanmar, Laos, Thailand, Vietnam, India, Nepal, and China (Sichuan) (Mazur 2011); Philippines (new record).

#### Biology.

*Atholustorquatus* (Marseul, 1854) were collected within the dung of cows located in a higher elevation and semi-forested area. The substrate also differs from *A.coelestis* (Marseul, 1857), as *A.torquatus* (Marseul, 1854) was typically observed in soggy, moist dung.

#### Remarks.

*Atholustorquatus* (Marseul, 1854) is a quite variable species regarding the external subhumeral stria on its elytra, either clearly marked or totally absent. This character is also mentioned by Desbordes (1917) who mentions the stria can be aberrant. Although the type specimen of *A.torquatus* (Marseul, 1854) according to the original description possesses no external subhumeral stria, we have examined one specimen with the subhumeral stria present. This corresponds to Desbordes’ (1917) observation. Our observations confirm the variability of this character among specimens ranging across Continental as well as Insular Southeast Asia. On the other hand, male and female genitalia exhibit little variation. We therefore propose to drop the external subhumeral stria as the primary key character for delimiting this species from others.

### ﻿ Atholus pirithous

Taxon classificationAnimaliaColeopteraHisteridae

(Marseul, 1873)

C9A24D84-EC42-57E5-A9AA-8C9D1560DAF9

Figs 4 , 44–49

Hister pirithous Marseul, 1873: 224 [Japan: Hiogo and Nangasaki].
Hister (Atholus) pirithous : Bickhardt 1910: 54 [catalogued]; 1913: 173; 1917: 194 [catalogued]; Desbordes 1919: 400 [Tonkin]; 1921: 10; Kamiya and Takagi 1938: 31 [listed]; Ôsawa and Nakane 1951: 7.
Atholus pirithous : Lewis 1906, 402; 1915, 55 [Formosa = Taiwan]; Nakane 1981: 10; Mazur 1984: 215; 1997: 132; 2009: 115; 2011: 106 [catalogued]; Mazur et al. 2014: 1269.
Atholus (Euatholus) pirithous : Kryzhanovskij and Reichardt 1976: 390; Hisamatsu and Kusui 1984: 23; Hisamatsu 1985: 223, pl. 41, fig. 19 [key; noted; image]; Ôhara 1993: 141–147; 1994: 138; 1999: 110 [Japan]; 1999b: 36 [Taiwan].
Hister reitteri Bickhardt, 1918: 231 [Japan]; synonymized by Reichardt 1930: 48; Kamiya and Takagi 1938: 31 [listed].
Hister pirithous ab. reitteri : Reichardt 1930: 48.

#### Specimens examined.

Seven specimens of undetermined sex. **Luzon Island**, Laguna, northern Lucena, Kinabuhayan, 7 exs. [IC-21-47], 1994-V-VI, N. Monreal leg.

#### Diagnosis.

*Atholuspirithous* (Marseul, 1873) is generally recognized for its light excavation in the area behind the anterolateral angle of the pronotum.

#### Distribution.

Japan, Russia: Far East, China (Guandong, Shanghai), Korea, Taiwan, Vietnam, Nepal, Oman (Mazur 2011); Philippines (new record).

#### Biology.

Unknown.

#### Remarks.

All seven examined individuals of *Atholuspirithous* (Marseul, 1873) (Fig. 4) lack internal subhumeral stria, but traces of dots and short lines can be observed in the apical end. The outer apical protibial tooth of this species is moderately prominent, topped by three denticles. The total number of protibial denticles on the outer margin is ten, one on the inner apical angle (Figs 48, 49), compared with the Japanese specimens described that bore only nine denticles (Ôhara, 1993), but the outer sublateral margin of the Philippine species has only four denticles; when compared to Ôhara (1993) who observed five to six denticles. Moreover, all examined specimens lost their genitalia prior to examination.

**Figures 44–49.**
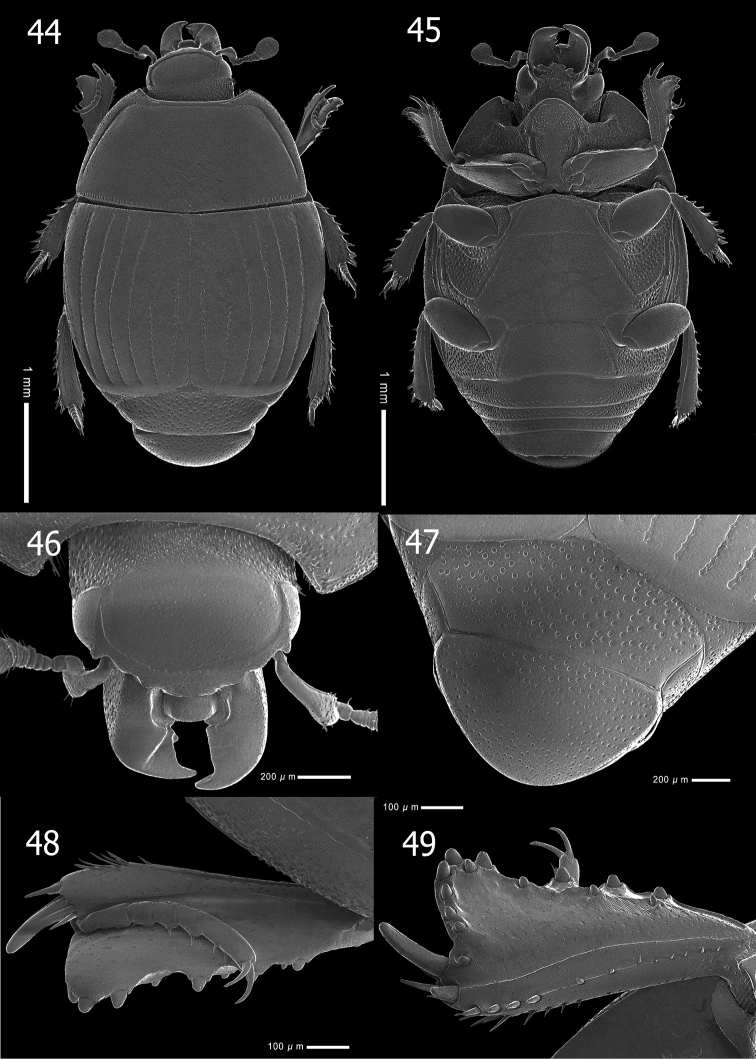
*Atholuspirithous* (Marseul, 1873) [IC-21-47] **44** habitus, dorsal view **45** ditto, ventral view **46** head, dorsal view **47** propygidium and pygidium **48** protibia, dorsal view **49** ditto, ventral view.

### ﻿ Atholus nitidissimus

Taxon classificationAnimaliaColeopteraHisteridae

Desbordes, 1925

A7A3931E-063A-533E-9D62-93C74FFDCD51

Figs 5 , 50–53

Atholus nitidissimus Desbordes, 1925: 87 [**Leyte Island**]; Mazur 1984: 215; 1997: 131; 2011: 105 [catalogued].

#### Specimens examined.

Two syntypes of undetermined sex housed in MNHN have been examined by N. Dégallier. The following re-description is based on images provided by him.

#### Diagnosis.

This species is easily distinguished by its almost circular body and absence of sutural elytral striae. Judging by the images of two examined syntypes, this species is clearly distinct in its pattern of dorsal elytral striation, differing from other species by the absence of the fifth or sutural elytral striae. *Atholusnitidissimus* Desbordes, 1925 (Fig. 5) is similar to *A.coelestis* (Fig. 2), albeit it is comparatively smaller in size than other species examined.

#### Re-description.

***Body* (Fig. 5) *length***: PEL: 2.15 mm; APW: 0.85 mm; PPW: 1.75 mm; EL: 1.15 mm; EW: 1.95 mm. Body almost circular, convex, and black; tibiae and antennae rufous.

**Figures 50–53.**
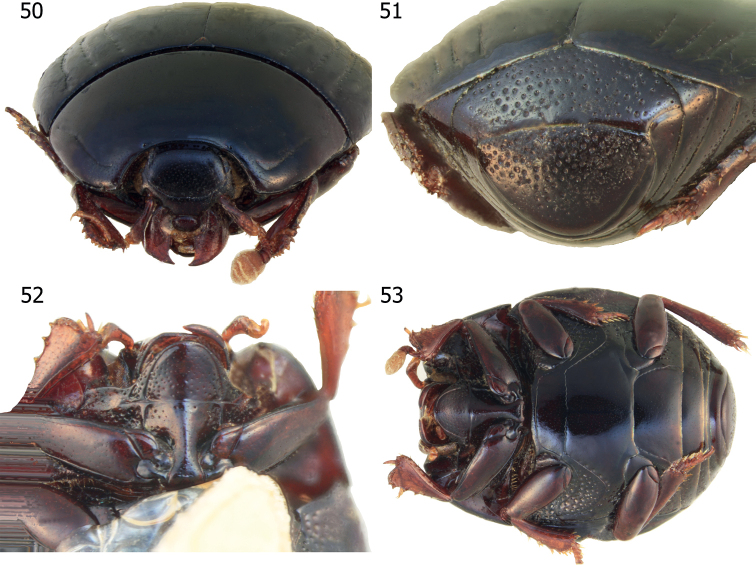
*Atholusnitidissimus* Desbordes, 1925 **50** anterior view **51** ditto, caudal view **52** prosternal process **53** habitus, ventral view.

***Head***: clypeus (Fig. 50) slightly crenate on anterolateral margin, apical margin projecting; frontal stria round, complete, and moderately impressed; eyes clearly visible dorsally; mandibles with rounded outer margin curved inwardly; mandibular apex acutely pointed.

***Pronotum***: marginal pronotal stria (Fig. 50) laterally complete, continuous onto the apical angle and behind head; lateral pronotal stria moderately impressed; apical end shortened and bent inwardly; lateral portion rather distant from margin; its basal end obsolete on basal sixth of pronotal length.

***Elytra***: external and internal subhumeral striae absent (Fig. 5); oblique humeral elytral stria lightly impressed on basal third; dorsal elytral striae 1–3 complete; elytral stria 4 present on apical half or shorter; elytral stria 5 either absent or very short; sutural elytral stria absent.

***Propygidium and pygidium***: propygidium (Fig. 51) moderately covered with coarse, round, and shallow punctures; interspaces with fine punctations; pygidial punctures similar to those of propygidium, albeit slightly denser.

***Prosternum***: anterior margin of prosternal lobe (Fig. 52) round; medio-apical end ascending; marginal prosternal stria impressed, shortly interrupted medially; short striae present on both baso-lateral ends; prosternal lobe with several punctures alongside marginal prosternal stria on both sides; entire disk covered with finer punctures; prosternal suture moderately impressed; prosternal process with few fine punctures; lateral sides descending; lateral prosternal striae deeply impressed; basal half of prosternal process narrow.

***Meso- and metaventrite***: anterior margin of mesoventrite (Fig. 53) truncate; marginal mesoventral stria complete; meso-metaventral suture clearly impressed, complete and carinate; lateral metaventral stria moderately impressed, carinate, extending obliquely and posteriorly, united with oblique humeral stria that inwardly extends from metaventro-metepisternal suture; post-mesocoxal stria extending posteriorly, strongly curved along the posterior mesocoxal margin, almost attaining the metaventro-mesepimeral suture; punctation of intercoxal disk of metaventrite similar to that of mesoventrite; longitudinal suture of metaventrite lightly impressed; lateral disk of metaventrite moderately covered with large, round, shallow punctures.

***Legs***: posterior surface of protibia (Figs 52, 53) flattened and dilated; outer lateral margin with four teeth, topped with minute denticles.

#### Distribution.

Endemic to the Philippines (Mazur 2011).

#### Biology.

Unknown.

### ﻿ Atholus bakeri

Taxon classificationAnimaliaColeopteraHisteridae

(Bickhardt, 1914)

75C9F8D8-3E37-5EE5-A522-75A7A0FB31A7

Figs 6 , 54–57

Hister bakeri Bickhardt, 1914: 428 [Luzon Island].
Hister (Atholus) bakeri : Bickhardt 1917: 193 [catalogued].
Atholus bakeri : Bickhardt 1914: 428; Mazur 1984: 211; 1997: 129; 2011: 103 [catalogued].

#### Specimens examined.

1 syntype [**Luzon Island**] based on images, “*Atholusbakeri* n. sp. Bickh. / Los Banos, / P.I., Baker. / 1639” [sex undetermined, measurements not available] (MNHUB).

#### Diagnosis.

This species has lateral pronotal striae interrupted in the anterolateral angle, and strong punctations on its entire pygidium. According to the original description of Bickhardt (1914), *Atholusbakeri* (Bickhardt, 1914) is most similar to *A.torquatus* (Marseul, 1854) except that the propygidium and pygidium of *A.bakeri* (Bickhardt, 1914) are strongly punctate. However, based on the syntype observed, the punctation is not as prominent as described and, in fact quite similar to that of *A.torquatus* (Marseul, 1854). The punctures are finer towards the apical end of the pygidium. Another distinguishable feature of *A.bakeri* (Bickhardt, 1914) is the medially straight frontal stria, while in *A.torquatus* (Marseul, 1854) it is weakly bent inwardly. However, several studied individuals of *A.torquatus* (Marseul, 1854) likewise seem to have their frontal stria medially straight.

#### Re-description.

***Body*** (Fig. 6) oval, moderately convex and black; tibia and antenna rufous.

**Figures 54–57.**
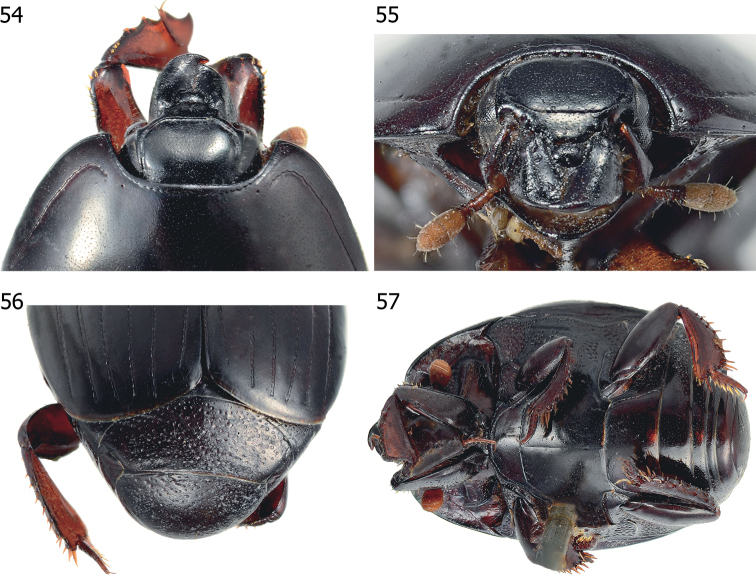
*Atholusbakeri* (Bickhardt, 1914) **54** head and pronotum, dorsal view **55** head, frontal view **56** propygidium and pygidium **57** habitus, ventral view.

***Head***: clypeus (Figs 54, 55) slightly crenate on anterolateral margin, apical margin slightly extended; frontal stria medially straight, complete, moderately impressed; eyes large, convex, clearly visible dorsally; mandibles with rounded outer margin curved inwardly; sub-apical tooth on left mandible large; mandibular apex acutely pointed.

***Pronotum***: marginal pronotal stria (Figs 54, 55) laterally complete, continuous onto apical angle and crenate behind head; lateral pronotal stria moderately impressed, slightly crenate; apical end shortened and bent inwardly in a curved hook; lateral portion rather distant from margin; its basal end abbreviated from basal fifth of pronotal length.

***Elytra***: elytral epipleuron (Fig. 57) with few coarse punctures on apical half; marginal epipleural stria present on apical half; marginal elytral stria (Fig. 6) complete, slightly impressed; external and internal subhumeral striae absent; oblique humeral stria lightly impressed on basal third; dorsal elytral striae 1–4 complete; dorsal elytral stria 5 and sutural elytral stria present on apical half; disk with fine punctures.

***Abdomen***: propygidium (Fig. 56) moderately covered with coarse, round, and shallow punctures; interspaces with fine punctations; pygidial punctations similar to those of propygidium, becoming sparser apically.

***Meso- and metaventrite***: anterior margin of mesoventrite (Fig. 57) outwardly arcuate; marginal mesoventral stria crenate and complete; meso-metaventral suture clearly impressed, complete, medially angulate; punctations of intercoxal disk of metaventrite similar to those of mesoventrite; longitudinal suture of metaventrite lightly impressed; lateral disk of metaventrite moderately covered with large, round, shallow punctures.

***Legs***: posterior surface of protibia (Fig. 57) flattened and strongly dilated; outer lateral margin with four weak, almost inconspicuous teeth, topped by minute denticles.

#### Distribution.

Endemic to the Philippines (Mazur 2011).

#### Biology.

Unknown.

#### Remarks.

The examined syntype of *A.bakeri* (Bickhardt, 1914) exhibits characters similar to a typical *A.torquatus* (Marseul, 1854). According to Desbordes (1917), *A.torquatus* (Marseul, 1854) and *A.bakeri* (Bickhardt, 1914) are very similar, being set apart by the pygidial punctation (strong in *A.bakeri* and apically finer in *A.torquatus*). Although the only examined specimen of *A.bakeri* (Bickhardt, 1914) possesses similar pygidial punctations to *A.torquatus* (Bickhardt, 1914), this character remains the primary distinction until further examinations of other types is established. The authors would also encourage a comprehensive observation of both male and female genitalia for future works.

## ﻿Discussion

Structures of the protibia in almost all Oriental species of *Atholus* were not described in detail in the original descriptions, particularly regarding the number and localization of denticles of protibia. In the previous works of Ôhara (1992, 1993, 1999b), the occurrence of denticles on designated margins such as lateral outer margin, anterior margin, and apical angle were described. However, since the protibial teeth of some *Atholus* species are not as strong as in others, it seems that the denticles on the apical angle may be ambiguously considered as denticles of either the apical margin, or of the outer lateral margin.

The gonocoxites of *A.philippinensis* (Marseul, 1854) and *A.torquatus* (Marseul, 1854) are relatively similar in their forms, appearing to be shovel-like in shape. We have observed this similarity with the gonocoxite of *Atholusbifrons* (Marseul, 1854) (dela Cruz and Ôhara 2022) from Ryukyus (Japan) and Borneo (Indonesia). On the other hand, the shape of the gonocoxite of *A.coelestis* (Marseul, 1857) is narrow and cone-like and becoming slenderer apically. Nevertheless, the number of spermathecal sacs (four) of *A.philippinensis* (Marseul, 1854), *A.coelestis* (Marseul, 1857), *A.torquatus* (Marseul, 1854), and even *A.bifrons* (Marseul, 1854) (dela Cruz and Ôhara 2022) is consistent among these species. Although we have not included this structure in the taxonomic key, since the female genitalia of other species examined were not available, the gonocoxite of *Atholus* might also become a useful tool for morphological diagnosis in the future.

*Atholus* species are generally widespread throughout the Oriental Region. A few species appear to be endemic to some regions such as *A.nitidissimus* Desbordes, 1925, only recorded so far from the island of Leyte in the Philippines, and *A.bakeri* (Bickhardt, 1914), reported only from Luzon Island hitherto. In this study, *A.coelestis* (Marseul, 1857) is revealed to be a ubiquitous species, spread across the islands of the Philippine archipelago. *Atholuspirithous* (Marseul, 1873) and *A.torquatus* (Marseul, 1854) are new records for Philippines. We examined six species of Philippine *Atholus* in this work; yet, we expect the number to rise in the future since the archipelago is situated in the vicinity of the Greater Sunda Islands in the Indonesian archipelago. It is therefore plausible that other species occurring there might also occur in the Philippines.

## Supplementary Material

XML Treatment for Atholus

XML Treatment for Atholus philippinensis

XML Treatment for Atholus coelestis

XML Treatment for Atholus torquatus

XML Treatment for Atholus pirithous

XML Treatment for Atholus nitidissimus

XML Treatment for Atholus bakeri
